# Dual-Scale Synergistic Design: Oriented Material Stiffness and Deposition Path Planning for Enhanced Performance in Large-Format Additive Manufacturing of Short Carbon Fiber Components

**DOI:** 10.3390/ma19112346

**Published:** 2026-06-01

**Authors:** Tao Yang, Chunjiang Zhao, Jianguo Liang, Wenzheng Li, Chen Wang, Zhangda Zhao, Kun Wang, Xiang Gu

**Affiliations:** 1 Engineering Research Center Heavy Machinery Ministry of Education, Taiyuan University of Science and Technology, Taiyuan 030024, China; 2 College of Mechanical Engineering, Taiyuan University of Science and Technology, Taiyuan 030024, China; 3 School of Intelligent Manufacturing Engineering, Shanxi University of Electronic Science and Technology, Linfen 041000, China; 4 College of Mechanical Engineering, Taiyuan University of Technology, No. 79, Yingze West Street, Taiyuan 030024, China; 5 Advanced Forming and Intelligent Equipment Research Institute, Taiyuan University of Technology, No. 79, Yingze West Street, Taiyuan 030024, China; 6 School of Rail Transit, Zhejiang Institute of Communications, Hangzhou 310000, China

**Keywords:** SCF/PA6 components, LFAM, deposition path planning, synergistic enhancement

## Abstract

**Highlights:**

- A novel process producing high-orientation and low-porosity tape-shaped beads.
- A dual-scale material-structure synergistic enhancement strategy.
- Peak load capacity surges to 549 N with 231% improvement.
- A new paradigm of multivariate coupling for multifunctional design.

**Abstract:**

Short carbon fiber-reinforced thermoplastic composites (SCFRTPCs) are widely employed in energy, aerospace and competitive sports due to their high specific strength/stiffness and design freedom. The Large Format Additive Manufacturing (LFAM) process, as an advanced technology for fabricating thermoplastic composite components, enables the rapid production of complex large-scale composite components and prototypes. Nevertheless, achieving satisfactory mechanical load-bearing performance remains a key challenge. To overcome this limitation, a methodology was developed for manufacturing short carbon fiber/Nylon 6 (SCF/PA6) composite components with programmable load-bearing performance via large-format additive manufacturing–compression molding (LFAM-CM). This process innovatively synergizes material stiffness enhancement with component deposition path planning, utilizing the high-orientation and low-porosity tape-shaped beads produced by LFAM to fabricate components. The experimental results demonstrate a peak load capacity of 549N, representing 33%, 231%, and 144% enhancements versus randomly oriented fiber, high-porosity, and non-path-planned components, respectively. Simultaneous meso- and macro-scale bearing performance analysis demonstrated the cross-scale synergistic enhancement effect of this process on component load-bearing capacity. Finally, a systematic analysis of energy dissipation, stiffness, and damage tolerance revealed the underlying mechanisms for enhanced load-bearing performance. This work establishes an expanded design paradigm where multivariate coupling replaces linear structure–property relationships, providing practical frameworks for the development of next-generation functionally graded components with tailored mechanical–electrical–thermal multifunctionality.

## 1. Introduction

Thermoplastic composites have emerged as highly sought-after lightweight engineering materials, not only due to their exceptional specific strength/stiffness and impact resistance, but primarily attributed to their unique combination of formability, recyclability, superior damping characteristics, and corrosion resistance. These distinctive advantages are driving the continuous expansion of their applications across aerospace, new-energy vehicles, wind turbine blades, marine vessels, and low-altitude aircraft systems [1,2,3,4,5]. SCFRTPCs utilize reinforcing fibers to enhance mechanical properties while maximizing the inherent advantages of thermoplastic matrices, including exceptional formability, high recyclability, and cost-effectiveness. These composites are extensively implemented in diverse manufacturing processes such as additive manufacturing [6,7], direct composite compression molding (DCCM), long-fiber thermoplastic direct injection/compression molding (LFT-D-IM/CM), and extrusion–compression molding (ECM). Components manufactured via these advanced techniques demonstrate significant commercial potential, particularly in lightweight structural applications [8,9,10,11]. However, there are still significant challenges in the rapid and low-cost preparation of large and complex components.

In recent years, LFAM, as a pellet-fed, screw-extrusion technology, has witnessed rapid advancement in additive manufacturing (AM), and demonstrates compelling speed, size, and cost advantages for manufacturing large-scale components [12]. Despite its advantages, LFAM faces critical challenges in manufacturing structural components. Current applications remain largely limited to non-/low-load-bearing scenarios, such as full-size automotive prototypes, naval mock-ups, and large-dimension tooling [13,14], with substantial research gaps persisting in load-bearing capacity. Key limitations include defect-induced mechanical degradation: similar to conventional AM, LFAM-processed materials exhibit significant porosity (>5 vol%), inter-bead voids, and insufficient fiber orientation, collectively compromising mechanical properties [15].

Oak Ridge National Laboratory (ORNL) pioneered BAAM-CM (Big-Area Additive Manufacturing with Compression Molding) technology, demonstrating that tamping compaction enhances strength and fiber-direction load transfer efficiency [16,17,18]; however, research on complex-load components remains in the evolutionary phase.

The research on the preparation process of the overall load-bearing performance of components has emerged in conventional 3D printing. Jikai Liu optimized deposition paths for fiber-reinforced brackets, achieving 40% higher load capacity [19]. Yuan Chen’s load-transfer path planning increased the peak load of composite parts 16.3-fold [20]. However, the application of LFAM technology to the above methods is lacking, and research in this area has only progressed to the compatibility stage of the material process [12], with limited exploration of load-driven path planning for short-fiber components.

In traditional forms of short carbon fiber-reinforced thermoplastic composite parts, performance enhancement is achieved through local reinforcement, such as the overmolding method, which necessitates a secondary manufacturing process. This increases production steps, prolongs cycle times, and raises energy consumption, exhibiting several drawbacks [21,22]. Therefore, identifying a process capable of directly achieving material enhancement during manufacturing is highly significant for improving the load-bearing capacity of the parts [23,24,25].

In one study, inspired by the microstructures of natural organisms, the key role of orientation control of the reinforcement phase in enhancing the overall performance of composites was discovered [26]. Helical fiber arrangements in the hammer-like stomatopod dactyl club achieves extraordinary damage tolerance [27], while out-of-plane-oriented calcite prisms in the nacreous layer of mollusk shells form self-protective architectures against external loads [28]. Precise microstructural control facilitates customized mechanical/thermal performance and enhances design flexibility. LFAM possesses a distinctive capability that addresses the preparation requirements of materials with orientation characteristics. The hierarchical multi-scale manufacture—spanning microstructural to mesoscale geometric features and macroscopic part dimensions [17]—imparts intrinsic material anisotropy [29].

**Figure 1:**
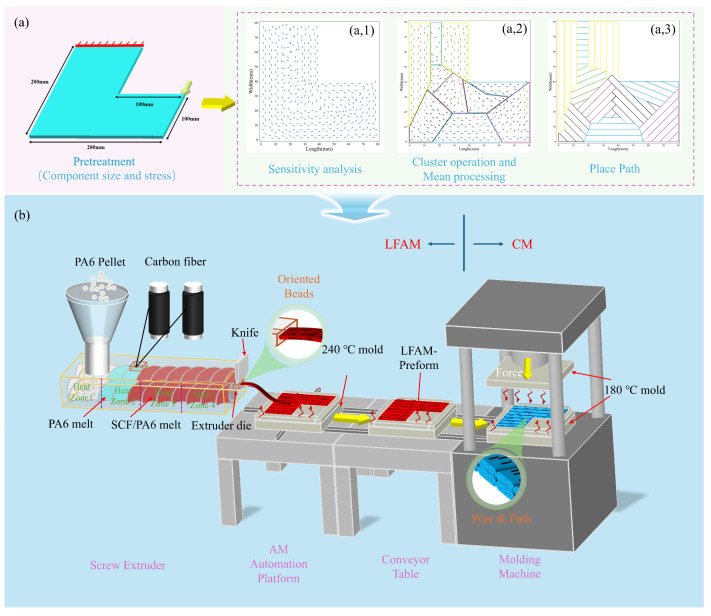
Process flow for manufacturing SCF/PA6 components. (**a**) Deposition path planning; (**a,1**) Sensitivity analysis; (**a,2**) Cluster operation and mean processing; (**a,3**) Place path; (**b**) manufacturing of components via LFAM-CM technique.

Consequently, enhancing the load-bearing capacity of short carbon fiber-reinforced thermoplastic (SCF/TP) composites necessitates novel process design to address insufficient fiber orientation, high porosity, inter-bead gaps, and inadequate component performance. This study pioneers an integrated LFAM approach (Figure 1) for SCF/PA6 composites, enabling tailorable mechanical properties through synergistic strategies: (1) stiffness-driven path planning under specific loads via CAE-based structural design; (2) material-level optimization via modified screw extrusion producing tape-shaped beads with high fiber orientation (>85% FOD); and (3) porosity and inter-bead gap suppression (<2.5 vol%) through compression molding (CM)-enabled consolidation. Through experimentation, the influences of material fiber orientation and porosity, as well as component path planning, on load-bearing capacity were demonstrated. Analysis via microscopic and macroscopic means revealed the synergistic enhancement mechanism of material and path factors within the component and the mechanism for load-bearing improvement.

## 2. Manufacturing of SCF/PA6 Composite Components

The “L-beam”, as a conventional load-bearing structure, is extensively utilized in aerospace, automotive, bicycle, and unmanned aerial vehicle (UAV) applications. This study focuses on a heavy-lift UAV’s “L”-shaped bracket under operational loading as a representative case to augment its load capacity.

### 2.1. Analysis of SCF/PA6 Composite Components

Enhancing the load-bearing capacity of SCF/PA6 composites necessitates a strategy: a manufacturing stage of component deposition path planning governed by the maximum stiffness criterion. This is achieved through a sensitivity analysis of fiber orientation distributions, clustering algorithms, and the statistical averaging of raw orientation data, as depicted in Figure 1a.

**Figure 2:**
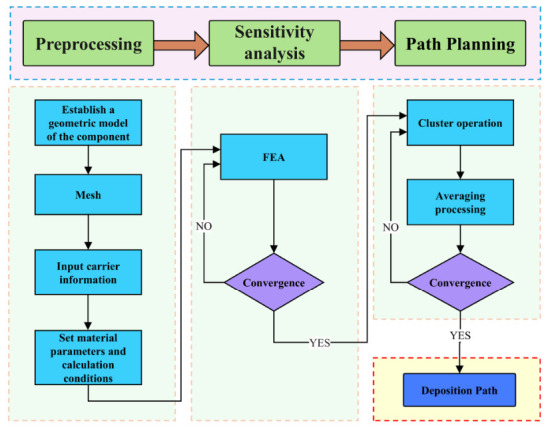
Workflow of path planning for SCF/PA6 composite components.

The workflow of path planning for SCF/PA6 composite components is shown in Figure 2, and includes three parts: preprocessing, sensitivity analysis, and path planning. The preprocessing includes establishing the geometric model of the component, meshing, inputting carrier information, and setting material parameters and calculation conditions. Path planning includes clustering operations and averaging processing. The final path planning is completed through Visio to refine the sedimentary path planning.

#### 2.1.1. Preprocessing of SCF/PA6 Composite Components

**Figure 3:**
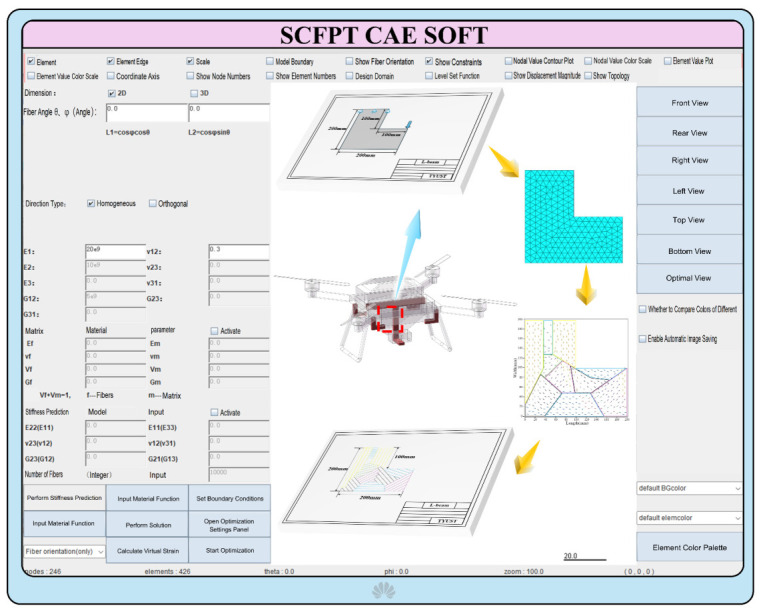
Finite element interface for CAE-driven analysis of SCF/PA6 components.

This study employs the Linux-compiled SFCANA featuring a modular architecture: GUI interface, finite element analysis, and post-processing modules. As shown in Figure 3, the integrated visualization window simultaneously renders (1) component geometry, (2) loading conditions, (3) fiber orientation optimization results, and (4) stiffness predictions. For SCF/PA6 components under specified loading regimes, the workflow executes (a) finite-element-based maximum stiffness analysis, (b) fiber orientation prediction via orientation tensor evolution modeling, and (c) sensitivity-driven fiber angle design. This integration method concentrates the preprocessing functions and has significant advantages in terms of iterative analysis cycle and overall design speed.

In the preprocessing stage, CAD engineering drawings are required to obtain the dimensional data of the “L-beam” component, including length, width, dimensions, and stress conditions. The geometric model of the SCF/PA6 composite component is then meshed. It is necessary to define material information and calculation conditions for the components. In this study, the most common load-bearing form of “L-beam” was taken as a specific load case. The long side of the component was set to be 200 mm, and the short side was set to be 100 mm. The load-bearing situation was that one short side was fixed, and a certain load-bearing capacity was applied to the other short side edge.

#### 2.1.2. Sensitivity Analysis of SCF/PA6 Composite Components

Fiber orientation sensitivity analysis was implemented via CAE finite element modeling to predict optimal fiber angles minimizing structural compliance, providing fundamental deposition path planning data (Figure 1a). Based on the specific load conditions of the SCF/PA6 composite components, using the maximum stiffness criterion, the minimum strain energy of the fibers in the unit is calculated from an energy perspective, and the fiber orientation sensitivity is calculated to obtain the optimized optimal fiber orientation angle. The optimization problem is represented using a unit orientation model (with orientation design variables $\theta_{e}$) [30]. Therefore, the optimization problem of orientation angle can be mathematically expressed as

$$\mathrm{find}\theta_{e}$$

(1) $$\min:J(\theta_{e})=C=2\int_{\Omega }E_{\mathrm{sed}}d\Omega$$

s.t.:K(θe)u=F−π2≤θe≤π2

where Ω represents the design domain; J is the objective function; C indicates total flexibility; K, u and F are respectively the overall stiffness matrix, displacement vector, and load vector; and $E_{\mathrm{sed}}$ represents the strain energy density. In the optimization problem studied in this section, since the angles of adjacent elements are not constrained, in order to establish a connection, it is necessary to establish an equilibrium equation using the displacement finite element method to establish angle constraints for adjacent elements using Equation (2), where the stiffness matrix is composed of a combination of element stiffness matrices, expressed as

(2) $$K_{e}=\int_{\Omega_{e}}b^{T}\left[\begin{array}{ccc} A_{e} & B_{e} & 0\\ B_{e} & D_{e} & 0\\ 0 & 0 & G_{e} \end{array}\right]bd\Omega_{e}$$

where b is the strain displacement matrix; and $A_{e}$, $B_{e}$, $D_{e}$ and $G_{e}$ represent the element membrane, membrane-bending coupling, bending, and transverse shear stiffness matrix, respectively. It can be calculated using the constitutive matrix as a reference [31].

Taking all unit directions as a vector field, a clustering algorithm is used to obtain its candidate angle set, and the optimal angle is filtered out to avoid local optima problems.

Fiber orientation can be described as

(3) $$\mathrm{fand}\theta_{e}(e=1,2\cdot \cdot \cdot N_{e})$$

(4) $$\max\;\prod =\sum_{e=1}^{N}f(\theta_{e})$$

where $\theta_{e}$ represents the local fiber angles of each unit; N represents the total number of units within a layer; $f(\theta_{e})$ represents the sensitivity of each unit; and Π represents the total strain energy of all units within a whole layer.

The periodic continuity of all terms in Equation (3) over [−90°, 90°] induces local optima issues. Fiber angle discretization was implemented uniformly, yielding sensitivity values for discretized elements. To determine optimal per-element angles, sensitivity values were sorted in descending order. Using the primary extremum as the reference, a threshold sensitivity was computed via an error coefficient β, as defined in Equation (5):

(5) $$\alpha_{e}^{th}=(1-\beta )\alpha_{e}^{\max}$$

where $\alpha_{e}^{\max}$ represents the maximum sensitivity of the e unit.

By using a one-dimensional clustering algorithm to screen the obtained data, two numerical families are obtained, and the sensitivity value of the fibers is used as the evaluation function to screen the candidate angles of the units. However, this method may result in multiple candidate angles appearing in some units. By using the sensitivity filtering similarity principle to process these units, the angle with the smallest difference from the surrounding angles is selected as the optimal angle based on the surrounding unit angles, solving the problem of continuity in orientation distribution. The final fiber orientation angle is shown in Figure 1(a,1).

#### 2.1.3. Path Planning of SCF/PA6 Composite Components

CAE-based analysis provides orientation data under specific loading conditions, yet raw angular values lack direct path-planning utility. Secondary clustering was implemented to categorize orientations into distinct angular clusters, with intra-cluster mean-value processing ensuring orientation uniformity. This methodology enables feasible deposition path generation for short-fiber thermoplastic composites.

To mitigate excessive angular dispersion in optimized fiber orientations, an angular deviation threshold ($\theta^{diff}$) was enforced prior to the weighted averaging of intra-cluster element angles. This ensured parallel vector alignment within clusters and completed secondary clustering, establishing well-defined deposition domains. Elemental optimal angles were combined with centroid coordinates, with deposition bead width calibrated against the cluster domain area to prevent domain–width mismatch (Figure 1(a,2)). The resultant clusters were color-coded; the angular deviation threshold $\theta^{diff}$ was 14°, enabling tool-path generation via CAD path plotting (Figure 1(a,3)).

### 2.2. Manufacturing Process of SCF/PA6 Composite Material Components

#### 2.2.1. Materials

The component consists of thermoplastic short carbon fiber composite materials, with the matrix material being nylon PA6 Ultramid^®^ B3S (supplied by BASF Germany via Suzhou Chuxuan Import & Export Co., Ltd., Suzhou, China), exhibiting a tensile strength of 75 MPa and preprocessed through drying at 80 °C for 8 h prior to use.

The reinforcement fiber utilizes SYT49S carbon fiber (tow specification: 12 K, average diameter: 7 µm) from Zhongfu Shenying Carbon Fiber Co., Ltd. (Lianyungang, China), demonstrating a tensile strength of 4900 MPa.

Manufacturing equipment includes a KTE-T-36B extruder (manufactured by Nanjing Kerk Extrusion Equipment Co., Ltd., Nanjing, China) featuring a screw length-to-diameter ratio of 40 (L/D = 40) and screw diameter of 36 mm, alongside a YQ32-400T four-column hydraulic press produced by Shandong Rugong CNC Machine Tool Co., Ltd. (Zaozhuang, China).

#### 2.2.2. Traceability Manufacturing of SCF/PA6 Composite Components

The preform manufacturing sequence is illustrated in Figure 1b.

Extruder heat zones were preheated to specified temperatures in Table 1; PA6 pellets were fed into the hopper, carbon fibers were introduced at Zone 2 for melt compounding, and the composite was extruded steadily at 40~50 kg/h.

**Figure 4:**
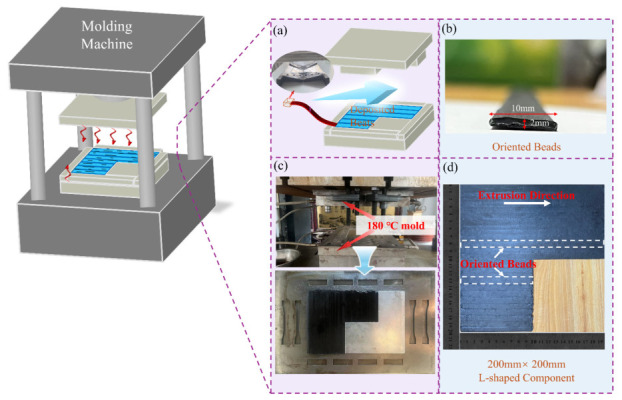
LFAM-CM process for SCF/PA6 composite components: (**a**) highly oriented preform manufacture; (**b**) compression mold design; (**c**) preform consolidation under pressure; (**d**) final SCF/PA6 composite components.

For orientation control, a wedge-shaped convergent flow channel (L × W: 12 × 2 mm) replaced conventional circular dies, inducing shear-driven fiber alignment along the extrusion direction shown in Figure 4a, thereby mitigating core-region misalignment. The highly oriented tape-shaped beads were produced as shown in Figure 4b.

Automated deposition was employed: die-cut oriented bead sizing; 240 °C constant-temperature mold on a programmable automated platform; path-synchronized material deposition.

Post-deposition compression molding was executed as shown in Figure 4c: mold transfer to molding machine; 180 °C mold temperature maintenance; compression at 7 MPa; 30 min isothermal curing. The demolded SCF/PA6 component underwent flash removal followed by 8-h annealing at 180 °C in vacuum oven, producing highly oriented, low-porosity composites via path-planned manufacturing (Figure 4d) [18,32].

## 3. Microstructure and Mechanical Characterization

**Figure 5:**
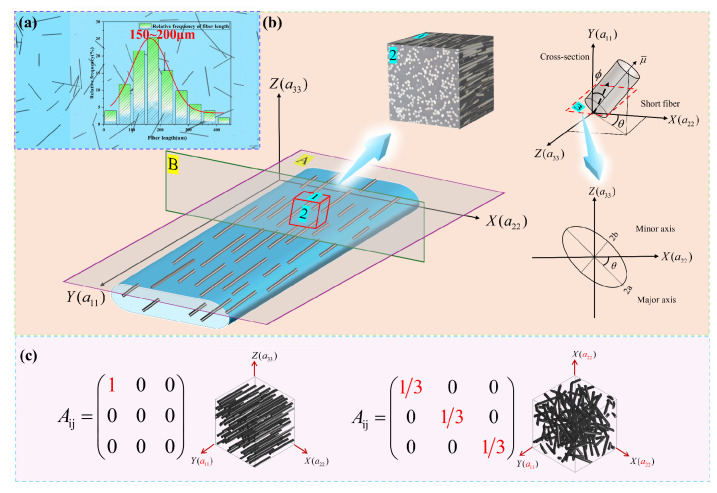
Characterization methods for fiber length and orientation: (**a**) Fiber length distribution diagram of SCF/PA6 composite material; (**b**) Spatial computation methodology for fiber orientation analysis in SCF/PA6 composites; (**c**) schematic diagram of fiber orientation degree.

The performance of SCF/PA6 components is influenced by factors such as deposition path, fiber length, fiber orientation, and porosity. Therefore, it is necessary to characterize the fiber length (Figure 5a), fiber orientation, porosity, and deposition path of the components after manufacturing and testing. This is of great significance for understanding the internal fiber situation of the materials and analyzing component damage and load-bearing performance.

### 3.1. Characterization of Fiber Orientation in SCF/PA6 Composite Materials

#### Fiber Orientation Characterization Protocol for SCF/PA6 Composites

Specimen manufacturing: sectioned 4 × 4 × 2 mm samples from molded composites mounted at the base of the embedding mold with epoxy resin (Component A/B = 2:1 ratio), cured for 20 min under ambient conditions.

Grinding was carried out using 1000-grit and 1200-grit SiC abrasive papers (Hangzhou Jingjing Testing Instrument Co., Ltd., Hangzhou, China), followed by sequential mechanical polishing using 3 μm polycrystalline diamond suspension and 0.05 μm alumina suspension (Hangzhou Jingjing Testing Instrument Co., Ltd., Hangzhou, China) on an automated polisher. The polishing parameters were maintained at 150 rpm with 20 N force for the diamond suspension and 150 rpm with 15 N force for the alumina suspension, each applied for 30 min.

Microstructural imaging: fiber orientation pattern images were captured at critical regions using a Leica DM6 microscope (Zhonghui Laibo (Beijing) Instrument Co., Ltd., Beijing, China) (Figure 5b). Quantified orientation was observed via tensor analysis (Figure 5c).

Fiber orientation analysis was performed by first extracting the elliptic parameters of composite cross-sections via MATLAB R2024a image processing, followed by conversion to spatial orientation tensors. The spatial relationship between the short-cut carbon fibers in the highly oriented extruded beads and the cross-section of the composite material sample, as well as the spatial shape and position relationship of individual fibers, are shown in Figure 5b. The ellipse orientation angle correspondence is presented, where major (2a) and minor (2b) axes define fiber ellipticity, with θ and ϕ denoting in-plane and out-of-plane inclination angles, respectively. These parameters were statistically analyzed across all fibers to derive the cross-sectional orientation tensor expressed in Equation (6) [33,34].

(6) $$A_{\mathrm{ij}}=\left(\begin{array}{ccc} a_{11} & a_{12} & a_{13}\\ a_{21} & a_{22} & a_{23}\\ a_{31} & a_{32} & a_{33} \end{array}\right)=\left(\begin{array}{ccc} \sin^{2}\phi \cos^{2}\theta & \sin^{2}\phi \sin\theta \cos\theta & \sin\phi \cos\phi \cos\theta \\ \sin^{2}\phi \sin\theta \cos\theta & \sin^{2}\phi \sin^{2}\theta & \sin\phi \cos\phi \sin\theta \\ \sin\phi \cos\phi \cos\theta & \sin\phi \cos\phi \sin\theta & \cos^{2}\phi \end{array}\right)$$

where $a_{11}$ represents the fiber arrangement in the extrusion direction of composite materials, i.e., the *Y*-axis; $a_{22}$ represents the arrangement of fibers in the width direction of composite materials, i.e., the X-axis; and $a_{33}$ represents the arrangement of fibers in the height direction of composite materials, i.e., the *Z*-axis.

The fiber orientation in the article is arranged along the extrusion direction, with a focus on discussing the numerical value of the fiber orientation tensor along the *Y*-axis ($a_{11}$), as the value approaching 1, indicates preferential fiber alignment along the extrusion direction (Figure 5c), whereas random fiber distribution yields comparable values for $a_{11}$, $a_{22}$ and $a_{33}$ [35]. Following single-fiber characterization, the statistical analysis of bulk fiber arrangement was performed. Considering the higher probability of fiber cross-section interception for fibers perpendicular to the cutting plane compared to parallel-oriented fibers, a weighting function was introduced during data correction in Equation (7), where $N_{i}$ denotes the weighting factor for the n-th fiber.

(7) $$N_{i}=\frac{1}{\cos\theta_{i}}$$

As shown in Equation (8), $a_{ij}^{\prime }$ represents the corrected fiber orientation tensor.

(8) $$a_{ij}^{\prime }=\frac{\sum_{k}N_{k}a_{ij}}{\sum_{k}N_{k}}$$

### 3.2. Characterization of Pore in SCF/PA6 Composite Materials

**Figure 6:**
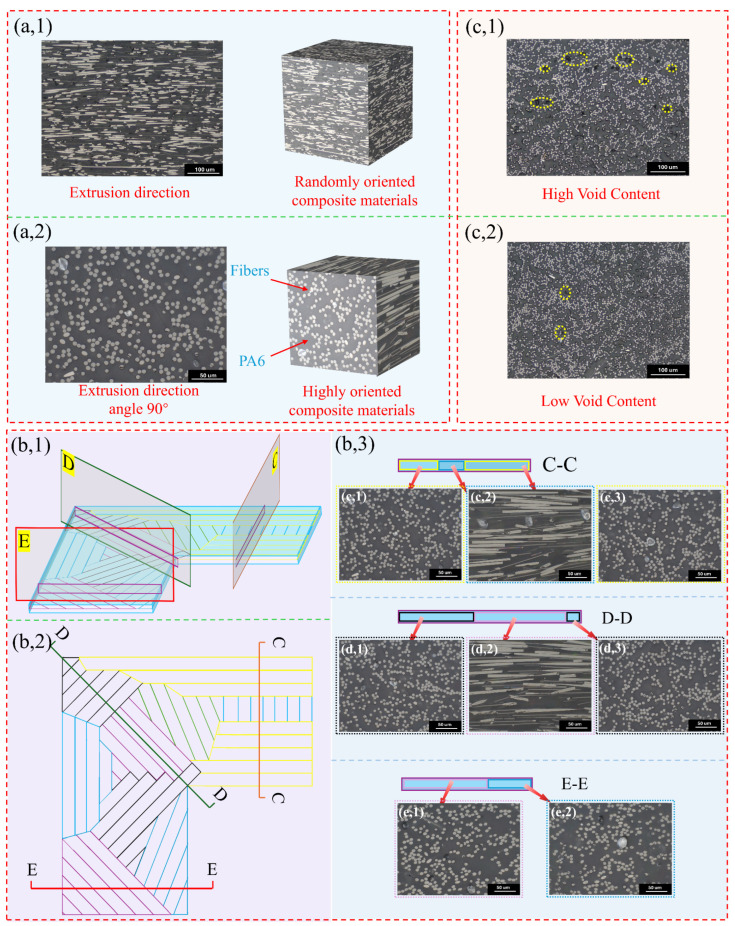
Characterization results of fiber orientation, porosity, and sedimentation resistance: (**a,1**) three dimensional orientation diagram of randomly oriented short carbon fiber composite materials; (**a,2**) three dimensional orientation diagram of highly oriented short carbon fiber composite materials; (**b,1**) characterization of sedimentation path and cross-sectional position of SCF/PA6 composite components (**b,2**) characterization of sedimentation path of SCF/PA6 composite components and cross-sectional top view position; (**b,3**) ((**b,3**) include (**c,1**)–(**c,3**), (**d,1**)–(**d,3**), (**e,1**) and (**e,2**)) SEM image of the deposition path characterization location of SCF/PA6 composite components; (**c,1**) SEM image of high porosity SCF/PA6 composite component material; (**c,2**) SEM image of low porosity SCF/PA6 composite component material.

The porosity characterization of the SCF/PA6 composite material was carried out by the method shown in Figure 6(c,1),(c,2): (1) preparing polished cross-sections by mounting specimens, (2) capturing pore morphology images using a Leica optical microscope (Zhonghui Laibo (Beijing) Instrument Co., Ltd., Beijing, China), (3) identifying porosity features through FIJI ImageJ v2.9.0 analysis, and (4) consolidating data from 10 images per sample to determine the final porosity values.

### 3.3. Characterization of Deposition Path of SCF/PA6 Composite Components

The fiber paths in composite components are engineered along deposition paths and formed via compression molding, though resin flow during processing inevitably perturbs fiber alignment. This necessitates establishing fiber orientation characterization points at critical design locations to quantify actual post-CM fiber orientations, as demonstrated in Figure 6(b,1)–(b,3).

As illustrated in Figure 6(b,1), three spatial sections (C, D, E) were designated on the component. Figure 6(b,2) displays the corresponding cross-sections (C-C, D-D, E-E) along these characterization paths: (1) C-C spans three zones with identical 90° fiber orientation angles in two zones and 0° in the central zone; (2) D-D exhibits identical orientation distribution to C-C; and (3) E-E covers two zones with fiber orientation angles of 135° and 60° respectively. Figure 6(b,3) quantifies the post-CM-process fiber orientations via ImageJ Orientation J analysis, where the in-plane inclination angles (hereafter termed “orientation angles”) were derived computationally.

### 3.4. Characterization of Load-Bearing Performance of SCF/PA6 Composite Components

**Figure 7:**
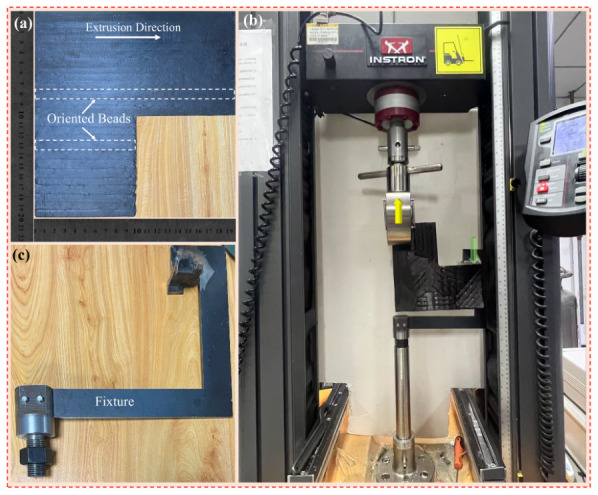
Load-bearing performance testing of SCF/PA6 composite components: (**a**) dimensional specification of SCF/PA6 composite components; (**b**) load-bearing performance testing of SCF/PA6 composite components; (**c**) fixture of SCF//PA6 composite component.

Load-bearing performance testing of SCF/PA6 composite components was conducted on an INSTRON 5969 universal testing machine (Instron (Shanghai) Testing Equipment Trading Co., Ltd., Shanghai, China) (Figure 7b), with distinct load conditions applied through the coordinated operation of the tensile rig and custom fixtures. Figure 7a depicts the specific dimensions of the manufactured component, while Figure 7c illustrates the custom fixture designed to simulate the specific loading conditions. The specimen was gripped at the upper end of the tensile testing machine to simulate the fixed end, with the fixture secured to the lower end. During the upward movement of the machine crosshead, the interaction between the pulled specimen and the fixed fixture applied the intended end-loading configuration. Tensile tests were conducted using a 50 kN load cell at a crosshead speed of 10 mm/min, generating load–displacement curves.

## 4. Results and Discussion

### 4.1. Load-Bearing Performance of SCF/PA6 Composite Components

**Figure 8:**
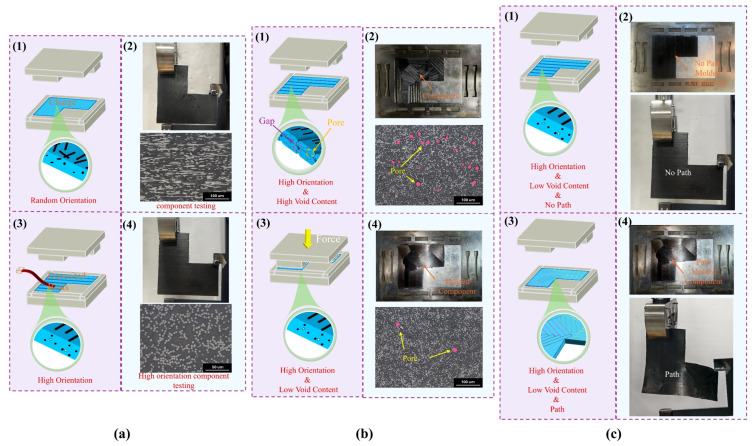
The influence of different processes on the load-bearing performance of SCF/PA6 composite components. (**a**) Manufacturing process of SCF/PA6 composites with different orientations; (**b**) manufacturing process of SCF/PA6 composites with different porosities and gaps; (**c**) manufacturing process of SCF/PA6 composites with different deposition paths.

This section describes the load-bearing performance of SCF/PA6 composite components. As shown in Figure 8, the load-bearing performance of composite components was studied through different processes, including fiber orientation, pores and gaps, and path planning of SCF/PA6 composite components.

#### 4.1.1. Effect of Fiber Orientation of SCF/PA6 Composites on Load-Bearing Performance

This section focuses on the influence of SCF/PA6 composite fiber orientation on material performance. It is well established that fiber alignment significantly enhances the load-bearing capacity along the axial direction, while its contribution in the radial direction is considerably lower and, under specific conditions, may even degrade performance, as confirmed in prior studies [36]. Consequently, to investigate the performance enhancement conferred by fiber orientation within the components, the load-bearing capacity of composites with randomly oriented fibers was compared against that of composites featuring highly oriented fibers, as presented in Figure 6(a,1),(a,2).

(1) Characterization of fiber orientation of SCF/PA6 Composites

Upon passing through a modified wedge-shaped die, the molten SCF/PA6 composite achieved directional alignment along the extrusion axis. This section comparatively analyzes non-oriented and die-oriented composites to characterize the wedge die’s influence on the orientation capability of tape-shaped beads.

Metallographic sections 1 and 2 from composite beads were examined, with results presented in Figure 6(a,1),(a,2). Figure 6(a,1),(a,2) display cross-sectional micrographs of randomly oriented and highly oriented composites. An analysis of Figure 8a reveals pronounced fiber orientation enhancement by the extrusion die: the non-die-oriented composite exhibited a fiber orientation factor of 0.45, whereas die-oriented specimens achieved 0.81. This validates the efficacy of the innovative wedge-shaped die in enhancing SCF/PA6 orientation during LFAM. The flattened die geometry mitigates the chronic issue of insufficient core shear in conventional composites that compromises orientation development [37].

(2) Load-bearing performance testing of SCF/PA6 composite components

Load-bearing performance tests of randomly oriented and highly oriented SCF/PA6 composites were carried out to study the effect of fiber orientation on the load-bearing performance of components.

**Figure 9:**
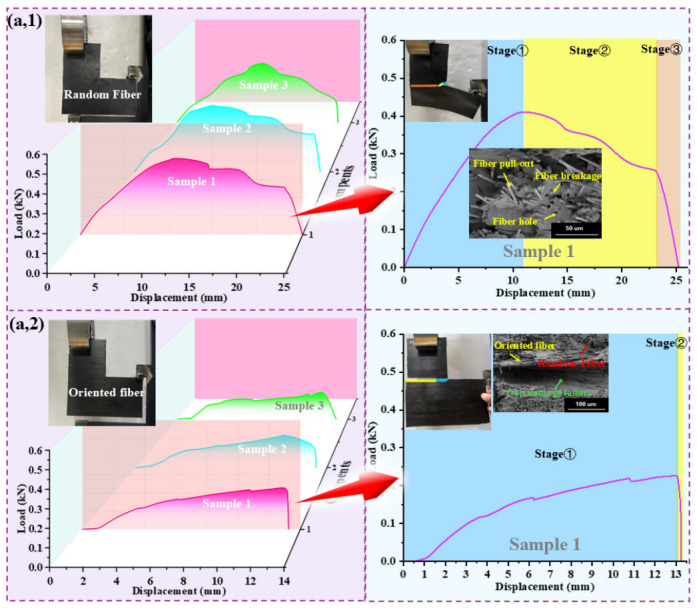

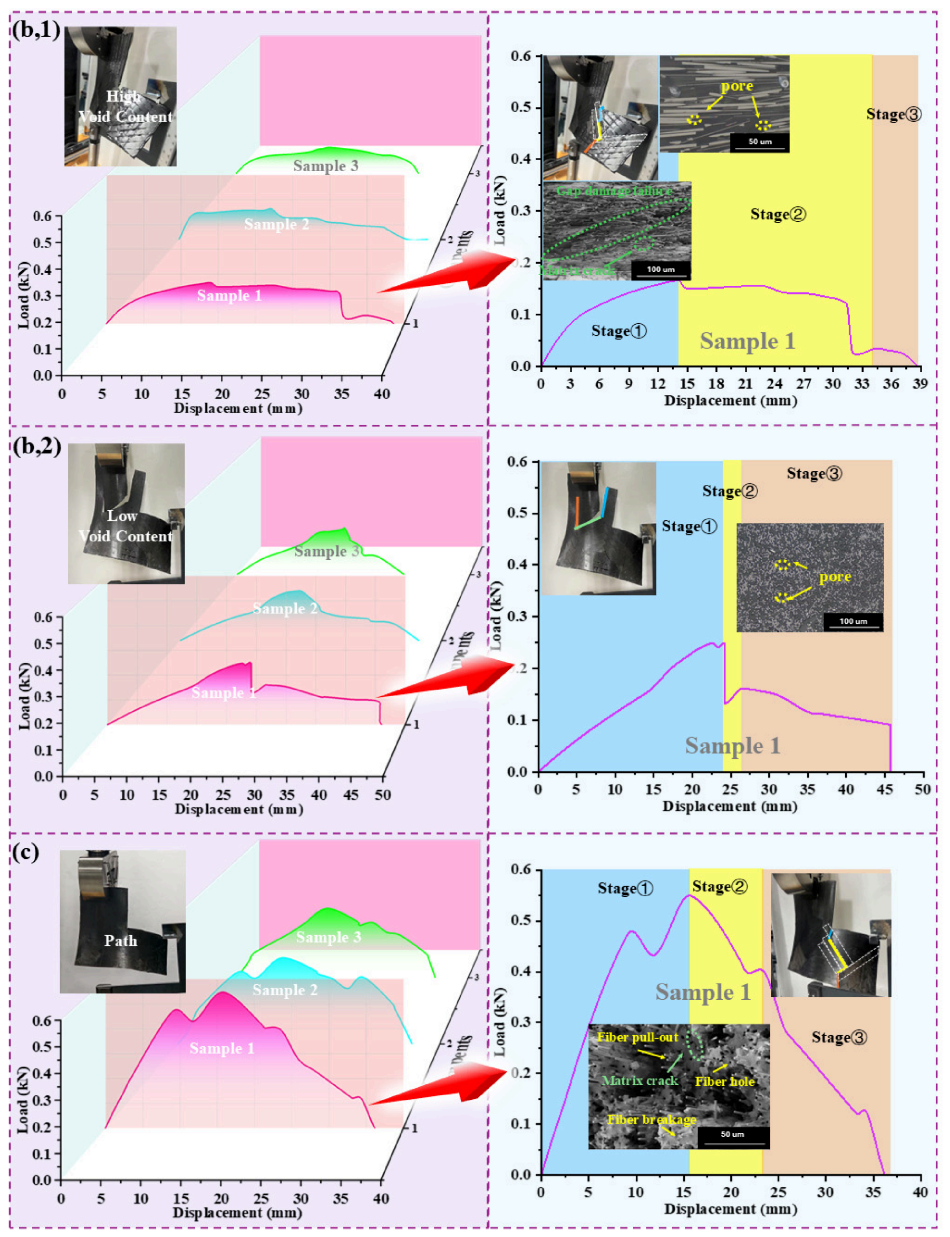
Load-bearing performance test of SCF/PA6 composite components prepared under different process conditions. (**a,1**) load-bearing performance testing of randomly oriented SCF/PA6 composite components; (**a,2**) load-bearing performance testing of composite components with oriented fibers; (**b,1**) effect of high porosity on load-bearing capacity of SCF/PA6 composite components; (**b,2**) effect of low porosity on load-bearing capacity of SCF/PA6 composite components; (**c**) effect of deposition path planning on the load-bearing capacity of SCF/PA6 composite components.

Figure 9(a,1) indicates a peak load of 413 N for randomly oriented components versus 225 N for highly oriented components, demonstrating superior load-bearing capacity in randomly oriented structures. However, an analysis of Figure 9(a,1),(a,2) reveals experimental limitations that preclude definitive conclusions regarding the relative load-bearing superiority of randomly oriented components. As evidenced in Figure 9(a,1), the tri-color sequence documents fracture propagation stages: First, stress concentration at the “L-beam” inner corner initiates crack nucleation under increasing load, with ordered damage extension at 413 N peak load; second, fiber-bridging mechanisms from randomly distributed fibers impede crack propagation, resulting in slow secondary crack formation; and third, accelerated crack expansion under continued tensile displacement leads to final fracture.

A highly oriented composite is shown in Figure 9(a,2). First, the stress concentration similarly induces crack initiation at geometric discontinuities; second, Rapid Mode-I brittle fracture occurs, causing abrupt load drop. SEM analysis reveals distinct reinforcement mechanisms between random and aligned fiber composites, a finding that has been repeatedly demonstrated in performance characterization studies in the fiber orientation regulation literature [36].

In the random composites shown in Figure 9(a,1), fiber bridging governs load-bearing capacity, with energy dissipated via fiber damage and pull-out. The same energy dissipation mechanism often occurs in the improvement of composite material performance [28]. Conversely, oriented fiber composites underperform in terms of their theoretical potential due to unoptimized fiber paths and inter-bead gaps. Figure 9(a,2) shows a hybrid microstructure from CM-induced fiber flow, explaining early plastic fracture and CM’s role in gap mitigation. Thus, strategic fiber path optimization is crucial to fully leverage alignment, ensuring that fibers bear axial loads. This will be validated in Section 4.1.3.

#### 4.1.2. Effect of Pores and Gaps of SCF/PA6 Composite Components on Load-Bearing Performance

(1) Characterization results of pores and gaps in SCF/PA6 composite components

The SCF/PA6 composite components have serious pore problems. The additive manufacturing process increases the spline gap, and the manufactured components are prone to failure. An efficient and rapid way to eliminate the porosity and gap of composites is the compression molding process shown in Figure 7b.

As evidenced in Figure 8c(1,2), SCF/PA6 composite components manufactured without compression molding exhibit an average porosity of 3.95%, whereas CM-processed counterparts demonstrate a reduced void fraction of 2.14% post molding. This represents a 45.82% reduction in porosity, confirming the significant efficacy of CM protocols for porosity suppression in SCF/PA6 composites.

(2) Load-bearing performance testing of SCF/PA6 composite components

Figure 9(b,1),(b,2) present comparative load–displacement curves for SCF/PA6 composite components manufactured without and with compression molding techniques, respectively. Specimens manufactured without CM (exhibiting elevated porosity) demonstrate a peak load-bearing capacity of 166 N, while CM-processed counterparts (with reduced porosity fraction) achieve 249 N. The damage evolution processes in composite components exhibit distinct three-stage mechanisms under loading, as illustrated in Figure 9(b,1),(b,2).

Figure 9(b,1) shows component damage evolution. First, the component undergoes a slow stage of destruction; upon reaching peak load-bearing capacity (166 N), rapid matrix shear tearing across the bead induces an abrupt load drop. Second, after brief propagation, the load-bearing capacity recovers transiently, followed by secondary matrix shear tearing and multiple crack deflection, resulting in multiple decline, after which cracks extend along gaps. Third, crack deflection along the specimen gaps drives sustained deterioration in load-bearing performance.

Figure 9(b,2) shows further component damage evolution. First, at peak load-bearing capacity (249 N), crack deflection is initiated, triggering a catastrophic load drop. Second, the transition from longitudinal to transverse propagation yields a brief load increase succeeded by gradual decline. Third, the shift from transverse to longitudinal extension along gaps precipitates a final abrupt load reduction.

These observations confirm that CM processing enhances load-bearing capacity by 50% through porosity and gap reduction (2.14% vs. 3.95%). There are pores at the fiber ends and matrix, as shown in Figure 9(b,1), and stress concentration leads to crack initiation, resulting in crack propagation across the beads. At the same time, due to the presence of gaps, the role of fibers cannot be fully utilized, and the matrix has a low load-bearing capacity, resulting in multiple crack deflection in the component without dissipating much energy. After the CM process shown in Figure 9(b,2), the porosity decreased and no crack propagation across beads occurred. Due to the fiber-bridging effect between beads, multiple crack deflection is accompanied by significant energy dissipation, resulting in a sharp decrease in bearing capacity. This reveals a positive correlation between mechanical performance and crack deflection frequency, suggesting that toughening mechanisms are activated through complex crack path tortuosity and demonstrating the significant effect of CM technology on the toughness and load-bearing capacity of the components. This process has been repeatedly demonstrated in automated fiber placement and has a significant effect on improving the mechanical properties of composite materials [15,19].

#### 4.1.3. Effect of Deposition Path Planning of SCF/PA6 Composite Components on Load-Bearing Performance

This study focuses on the influence of deposition path planning on mechanical performance while elucidating the underlying mechanisms of stress redistribution and fiber alignment optimization. The methodology combines finite element analysis with experimental validation to establish process–structure–property relationships.

(1) Characterization of deposition path planning of SCF/PA6 composites

The deposition path planning (path) characterization results of SCF/PA6 composite components are shown in Figure 6(b,1)–(b,3). The design angle and fiber orientation angle after the CM process were tested at three cross-sections (C-C, D-D, E-E), and the degree of influence of the CM process on the design path was finally calculated. The data comparison in Table 2 shows that the path was not significantly affected. These variations, though discernible, remain within acceptable process tolerances (<3° relative error), confirming the effective retention of the planned deposition path geometry.

(2) Load-bearing performance testing of SCF/PA6 composite components

As shown in Figure 9(a,2),c, the load-bearing capacity–displacement diagrams of the components without and with deposition path planning are presented, respectively. The peak bearing capacity of the components without deposition path planning reached 225 N, while that of the components with deposition path planning reached 549 N, an increase of nearly 144%.

As shown in Figure 9(a,2), the component without deposition planning undergoes a fracture failure process during the bearing capacity test, and the component undergoes Type I fracture. Without deposition planning, the component cannot fully exert its strengthening effect from fibers. For specific details, please refer to Section 4.1.1.

Figure 9c shows component damage evolution. First, the initial loading phase demonstrates stable load increase with minor peak fluctuations, followed by a 50 N load drop before reaching the maximum bearing capacity of 549 N, where catastrophic load reduction occurs upon first crack propagation across the bead. Second, the secondary phase shows temporary load recovery during major crack extension, abruptly terminated by crack deflection-induced stress redistribution. Third, the final failure phase features continued crack deflection with progressive stiffness degradation until complete structural collapse.

The deposition path planning components demonstrate 144% higher bearing capacity (Pmax = 549 N) than without deposition path planning components (Pmax = 225 N), quantitatively validating the critical role of deposition path planning optimization in load-bearing enhancement (Figure 9c vs. Figure 9(a,2)). The fracture diagram in Figure 9c shows fiber fracture, fiber pull-out, and matrix damage, indicating that, in the stress propagation path, the fibers and matrix dissipate a large amount of energy, improving the load-bearing capacity of the component. At the same time, after the CM process, the stress concentration problem caused by gaps and pores sharply decreases, ensuring the smooth loading path of stress.

Based on the above fracture analysis, different failure modes were revealed: (1) catastrophic fracture without secondary loading in components without deposition path planning (Figure 9(a,2)) versus (2) multi-stage failure with stable stiffness retention (initial phase), sequential crack deflection, and trans-bead crack propagation in planned components (Figure 9c), exhibiting superior damage tolerance through 2–3 load redistribution events. Multiple studies have demonstrated the effectiveness of damage tolerance in improving the load-bearing performance of composite components [26]. This performance synergy stems from the coupled design strategy combining load-case-specific path planning with highly oriented fiber architecture and the elimination effect of the CM process on stress, which collectively improve stress transfer efficiency. The demonstrated methodology provides a validated framework for the design of functionally tailored composite structures, particularly where damage-tolerant failure progression is required.

### 4.2. Load-Bearing Mechanisms

**Figure 10:**
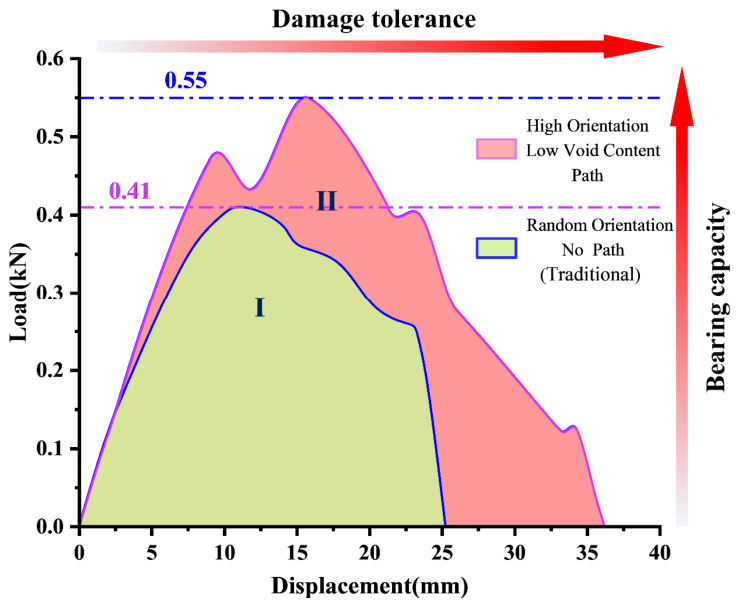
Comparison of power–displacement of composite components developed with new and traditional processes.

This study energetically evaluates SCF/PA6 composite component performance (Figure 10). A comparative analysis revealed superior load-bearing capacity in the novel-processed components versus conventional counterparts, with distinct advantages: (1) elastic deformation phase: enhanced stiffness and elastic energy absorption, indicating higher stability. Sequential load-bearing stages at elevated stress demonstrated staged stiffness activation, highlighting stiffness-oriented design efficacy. (2) Plastic fracture phase: substantially greater energy dissipation (plastic work) via organized sequential consumption (I vs. I + II), confirming improved fracture toughness.

The energy absorption mechanism manifests primarily through elastic potential energy storage during the elastic phase and fracture energy dissipation during failure, with structural stiffness governing the former and damage tolerance determining the latter. Our systematic investigation focuses on these two critical aspects to elucidate the enhanced load-bearing mechanisms: (1) the multi-scale stiffness synergy between composite architecture and highly oriented fiber bundles creates graded stiffness distribution; (2) the crack deflection and staged propagation mechanisms of cracks increase damage tolerance, as quantified through fracture surface analysis. These findings demonstrate that optimized stiffness hierarchy coupled with controlled crack path tortuosity synergistically enhances the composite’s energy absorption capacity.

#### 4.2.1. Structural Stiffness

This study employs structural stiffness optimization to enhance load-bearing performance through a dual-strategy approach. For global stiffness, the preparation process of carbon fiber composite components in this study illustrates stiffness performance pre-definition via fiber path planning guided by sensitivity analysis [30], where minimum strain energy criteria under specific loading conditions determine optimal fiber orientations. The efficacy of this planning is illustrated in Figure 9c, revealing a 144% increase in load capacity between path-planned and conventional specimens manufactured identically. Crucially, the path-planned components demonstrate superior elastic-stage stiffness (Figure 9c), attributable to strategic fiber orientation along primary load paths that promotes uniform stress distribution [36]. Structural redundancy is evidenced through residual post-instability stiffness [38]. Material-wise, the preparation process of carbon fiber composite components in this study confirms that highly oriented tape-shaped beads preserve intrinsic stiffness [39], while Figure 9(b,1) shows interfacial pores—elongated along fiber axes—as stress concentration sites at fiber–matrix interfaces [15,40]. The comparative fractography shown in Figure 9(b,1),(b,2) demonstrates that CM processes mitigate trans-bead crack propagation (Figure 9(b,1) vs. Figure 9(b,2)), enhancing strength and stiffness [41]. The synergistic integration of structural design with advanced material processing establishes a co-optimization framework for stiffness-engineered components.

In conclusion, SCF/PA6 composite structures manufactured via LFAM demonstrate validated stiffness-driven design efficacy [41,42,43]. By adopting maximum stiffness as the fundamental design criterion—achieved through highly oriented fiber architectures and CM processes—these components ensure efficient axial stress transfer while minimizing stress concentration, thereby enhancing global structural rigidity. This approach establishes a synergistic enhancement mechanism wherein load-path-planned path planning (tailored to specific service conditions) couples with high-orientation/low-porosity material engineering. The resultant programmable stiffness modulation enables co-optimized load-bearing performance, representing an editable LFAM structural design paradigm for advanced composites.

#### 4.2.2. Damage Tolerance

Figure 9(a,2) reveals rapid crack nucleation followed by unstable propagation and brittle fracture in the control specimens, indicating minimal energy absorption and low damage tolerance. In contrast, the SCF/PA6 composites exhibit enhanced damage tolerance through controlled crack deflection and staged propagation.

**Figure 11:**
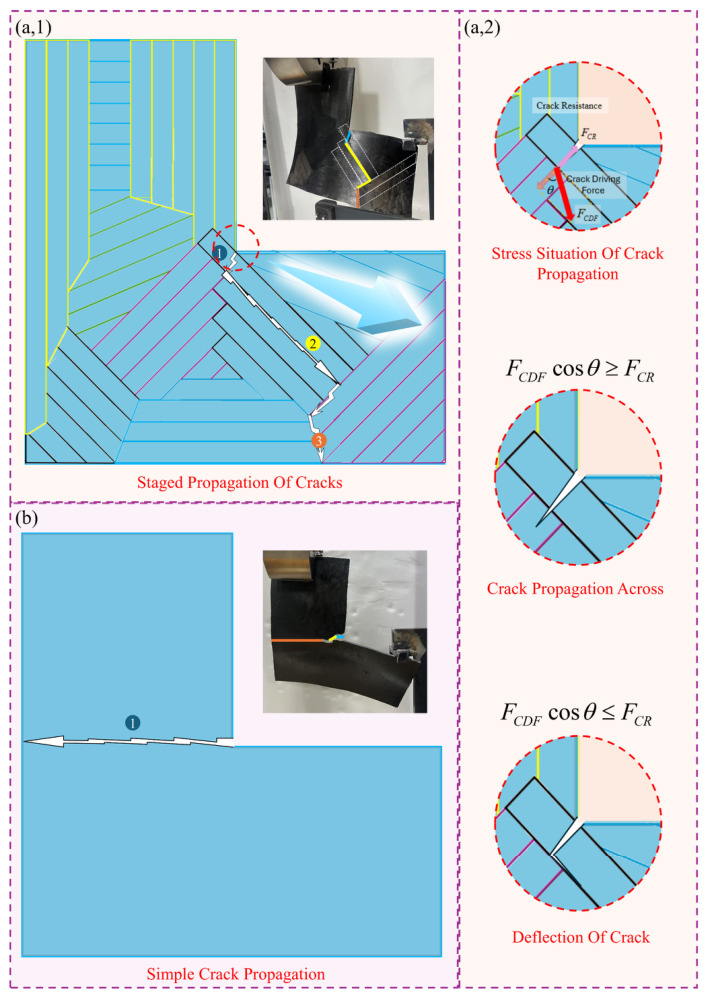
Damage mechanism diagram of SCF/PA6 composite component. (**a,1**) Damage failure evolution diagram of SCF/PA6 composite component; (**a,2**) mechanism diagram of trans-bead crack propagation and deflection; (**b**) damage failure evolution diagram of low-damage-tolerance SCF/PA6 composite components.

Crack deflection behavior is governed by three factors: interfacial effects, crack driving force ($F_{CDF}$), and propagation direction. As demonstrated in Figure 9(a,1), the absence of interfacial reinforcement induces stiffness-induced structural instability under in-service loading conditions, ultimately triggering catastrophic failure. Conversely, engineered specimens leverage fiber–matrix interfaces and micro-gaps to suppress catastrophic failure (Figure 9c): when $F_{CDF}$ < crack resistance ($F_{CR}$), crack tips deflect toward weak paths (Figure 11(a,2)), with deflection angle θ determining the energy dissipation efficiency [41]. This mechanism provides higher damage tolerance than conventional systems. Trans-bead crack propagation occurs when $F_{CDF}$ significantly exceeds $F_{CR}$ in Figure 9c, typically during stiffness transitions. Such events release substantial energy through matrix cracking/fiber pullout [42], and the sharp decline in the peak load-bearing capacity observed during testing can verify this assertion. Stage ② demonstrates nuanced behavior: despite small θ angles similar to trans-bead crack propagation events, reduced $F_{CDF}$ values promote deflection rather than trans-bead crack propagation. The transient load recovery observed stems from energy accumulation during deflection, while subsequent load decline corresponds to crack re-initiation. Fundamentally, transverse cracking correlates with high $F_{CDF}$ values and massive energy dissipation, while deflection requires balanced $F_{CDF}$–$F_{CR}$ relationships and favorable θ angles. This fracture mode modulation establishes a programmable damage tolerance mechanism in composites.

Secondary to this, staged crack propagation correlates with progressive damage resistance in components, fundamentally representing phased energy dissipation. As illustrated in Figure 11b, conventional components exhibit crack nucleation prior to yielding, demonstrating minimal load peaks before ultimate failure. This process displays smooth progression followed by abrupt load instability, leading to catastrophic failure. Conversely, Figure 11(a,1) illustrates components undergoing multiple high-amplitude load peaks during damage evolution, succeeded by progressive stepwise degradation. This behavior originates from path-planned components exhibiting unique structural performance: superior load-bearing capability during the elastic phase enables continuous strain energy accumulation until reaching critical stiffness thresholds. Post-ultimate-load, trans-bead crack propagation triggers significant load reduction, yet demonstrates substantially enhanced energy dissipation capacity and damage tolerance compared to those in Figure 11(a,1). Initial load reduction manifests energy absorption through fiber/matrix fracture mechanisms, while retained high residual strength facilitates crack deflection—observable as transient load recovery events [44,45,46]. After multiple damage cycles, structural integrity progressively degrades toward final failure. The combinatorial mechanism of alternating trans-bead propagation and crack deflection constitutes staged crack advancement [47]. Each load peak corresponds to either trans-bead propagation or deflection, collectively establishing phased energy dissipation. This intricate failure progression prevents catastrophic collapse while enhancing damage tolerance and load-bearing capacity [42].

In summary, energy dissipation during crack propagation is governed by damage tolerance mechanisms, primarily involving crack deflection (controlled by interfacial efficacy and crack-driving force/resistance balance) and staged propagation (modulated through structural damage resistance). Consequently, path planning, fiber orientation control, and CM processes critically synergize to enhance SCF/PA6 component performance. This integrated approach achieves distinctive structural stiffness and fracture toughness, ultimately elevating load-bearing performance.

## 5. Conclusions

In summary, this study successfully integrated predefined stiffness design with LFAM processes. Through innovative path planning coupled with highly-oriented, low-porosity composite architecture coupling design, this study manufactured SCF/PA6 components exhibiting tailored load-bearing performance.

Mechanical testing demonstrated exceptional capacity with a peak load of 549 N, representing 33%, 231%, and 144% improvements over randomly-oriented fiber, high-porosity, and non-path-planned counterparts, respectively. Mesoscopic and macroscopic examination of the fracture surfaces reveals that this performance enhancement originates from the coupled design strategy combining load-case-specific path planning with a highly oriented fiber architecture and the elimination effect of the CM process on stress, which collectively improve stress transfer efficiency.

The systematic investigation of damage evolution revealed that predefined deposition paths and optimized fiber-oriented composites effectively control both structural stiffness and damage tolerance. In terms of stiffness-programmable enhancement, the multi-scale stiffness synergy between path-planned components and highly-oriented fiber composites creates editable structural performance. For damage tolerance regulation, crack deflection and staged propagation collectively prevent catastrophic failure. Optimized stiffness hierarchy coupled with controlled crack path tortuosity synergistically enhances the composite’s energy absorption capacity.

These performance enhancements fundamentally originate from our innovative function-driven design methodology and composite manufacturing processes, highlighting the critical synergy between maximum-stiffness-based deposition path planning and highly-oriented, low-porosity composite components. The proposed component design and manufacturing framework enables unprecedented multidimensional design freedom, effectively transforming conventional linear structure–performance relationships into multimodal coupling effects across scales. This work demonstrates the immense potential of integrating predefined component design with advanced composite manufacturing (such as vibration characteristics, light weight, fatigue characteristics, and thermal conductivity), establishing practical foundations for manufacturing large-format heterogeneous functional components with programmable mechanical–electrical–thermal properties.

## Figures and Tables

**Table 1:** Processing parameters for manufacturing highly oriented discontinuous fiber composite beads.

Project	Numerical Value			
Extruder spindle speed (rpm)	150			
Ingredient screw speed (rpm)	2.5			
Fiber length (µm)	150~200			
Fiber quality fraction (%)	40			
Heat zone	1	2	3	4
Temperature (°C)	240	240	240	250

**Table 2:** Characterization results of fiber orientation angles before and after CM process for the deposition path of SCF/PA6 composite prefabricated components.

Cross-Sections	Design Orientation Angle			After CM		
C-C	90°	0°	90°	89.87°	0.87°	88.56°
D-D	90°	0°	90°	88.34°	0.54°	89.89°
E-E	135°		60	132.56°		61.35°

## Data Availability

The original contributions presented in this study are included in the article. Further inquiries can be directed to the corresponding authors.

## References

[1] Barnett P.R., Hmeidat N.S., Zheng B., Penumadu D. (2024). Toward a circular economy: Zero-waste manufacturing of carbon fiber-reinforced thermoplastic composites. npj Mater. Sustain..

[2] Huang C., Lv D., Zhu Y., Chen G., Chen M., Zhang Y., Han Y., Wu H. (2024). Influences of heat treatment on mechanical properties of SCF/PEEK composites in FDM-3D printing process with UV laser assistance. Polym. Compos..

[3] Liang J., Gu X., Liu J., Wu T., Zhang X., Zhao R., He L. (2025). Low-Velocity Impact of Short Carbon Fiber Reinforced Thermoplastic Sheet by Direct Composite Compression Molding. Polym. Compos..

[4] Wang Y., Ye H., He J., Ge Q., Xiong Y. (2024). Electrothermally controlled origami fabricated by 4D printing of continuous fiber-reinforced composites. Nat. Commun..

[5] Billah K.M.M., Kumar V., Rathod N., Phadatare A., Saha S., Smith T., Nuttal D., Kim S., Vaidya U., Hassen A.A. (2025). Fast-rate joining of thermoplastic composites using integrated additive manufacturing and compression molding process. J. Manuf. Process..

[6] Yu K., Dunn M.L., Jerry Qi H., Maute K. (2025). Recent advances in design optimization and additive manufacturing of composites: From enhanced mechanical properties to innovative functionalities. npj Adv. Manuf..

[7] Etemadi E., Zhang M., Li K., Bashtani M., Ho M.M.P., Tahir D., Hu H. (2023). Load-bearing characteristics of 3D auxetic structures made with carbon fiber reinforced polymer composite. Compos. Struct..

[8] Zhao R., Gu X., Wu T., Li Y., Zhao X., Li H., Liang J. (2024). Compression behavior of direct compounded compression molded short carbon fiber reinforced thermoplastic pyramidal lattice truss core. Compos. Part B Eng..

[9] Ning H., Lu N., Hassen A.A., Chawla K., Selim M., Pillay S. (2019). A review of Long fibre thermoplastic (LFT) composites. Int. Mater. Rev..

[10] Hu J., Zhan L., Yang X., Shen R., He J., Peng N. (2020). Temperature optimization of mold for autoclave process of large composite manufacturing. J. Phys. Conf. Ser..

[11] Park J., Cho C., Kim K.H., Kang J., Seo M., Choi B., Kim S.Y. (2025). Effect of plating thickness on PA6/nickel-plated carbon fiber composites manufactured using LFT pellets. Polym. Compos..

[12] Roschli A., Gaul K.T., Boulger A.M., Post B.K., Chesser P.C., Love L.J., Blue F., Borish M. (2019). Designing for Big Area Additive Manufacturing. Addit. Manuf..

[13] Ishikawa T., Amaoka K., Masubuchi Y., Yamamoto T., Yamanaka A., Arai M., Takahashi J. (2018). Overview of automotive structural composites technology developments in Japan. Compos. Sci. Technol..

[14] van de Werken N., Tekinalp H., Khanbolouki P., Ozcan S., Williams A., Tehrani M. (2020). Additively manufactured carbon fiber-reinforced composites: State of the art and perspective. Addit. Manuf..

[15] Al-Maharma A.Y., Patil S.P., Markert B. (2020). Effects of porosity on the mechanical properties of additively manufactured components: A critical review. Mater. Res. Express.

[16] Kumar V., Alwekar S.P., Kunc V., Cakmak E., Kishore V., Smith T., Lindahl J., Vaidya U., Blue C., Theodore M. (2021). High-performance molded composites using additively manufactured preforms with controlled fiber and pore morphology. Addit. Manuf..

[17] Duty C.E., Kunc V., Compton B., Post B., Erdman D., Smith R., Lind R., Lloyd P., Love L. (2017). Structure and mechanical behavior of Big Area Additive Manufacturing (BAAM) materials. Rapid Prototyp. J..

[18] Barakat A., Šeta B., Meraki Y., Talabi S.I., Chawla K., Spangenberg J., Kumar V., Hassen A.A., Vaidya U. (2025). Numerical framework for integrated additive manufacturing-compression molding (AM-CM) of thermoplastic composites. Compos. Part A Appl. Sci. Manuf..

[19] Liu J., Huang J., Yan J., Li L., Li S. (2022). Full sensitivity-driven gap/overlap free design of carbon fiber-reinforced composites for 3D printing. Appl. Math. Model..

[20] Chen Y., Ye L. (2021). Topological design for 3D-printing of carbon fibre reinforced composite structural parts. Compos. Sci. Technol..

[21] Boros R., Rajamani P., Kovács J. (2018). Thermoplastic Overmolding onto Injection-Molded and In Situ Polymerization-Based Polyamides. Materials.

[22] Murray J.J., Allen T., Bickerton S., Bajpai A., Gleich K., McCarthy E.D., Brádaigh C.M.Ó. (2021). Thermoplastic RTM: Impact Properties of Anionically Polymerised Polyamide 6 Composites for Structural Automotive Parts. Energies.

[23] Elkholy A., Rouby M., Kempers R. (2019). Characterization of the anisotropic thermal conductivity of additively manufactured components by fused filament fabrication. Prog. Addit. Manuf..

[24] Qu M., Nilsson F., Schubert D.W. (2018). Effect of Filler Orientation on the Electrical Conductivity of Carbon Fiber/PMMA Composites. Fibers.

[25] Zhou H., Liu H., Jiang Q., Kuang T., Chen Z., Li W. (2019). Effect of Process Parameters on Short Fiber Orientation along the Melt Flow Direction in Water-Assisted Injection Molded Part. Adv. Mater. Sci. Eng..

[26] Raney J.R., Compton B.G., Mueller J., Ober T.J., Shea K., Lewis J.A. (2018). Rotational 3D printing of damage-tolerant composites with programmable mechanics. Proc. Natl. Acad. Sci. USA.

[27] Weaver J.C., Milliron G.W., Miserez A., Evans-Lutterodt K., Herrera S., Gallana I., Mershon W.J., Swanson B., Zavattieri P., DiMasi E. (2012). The Stomatopod Dactyl Club: A Formidable Damage-Tolerant Biological Hammer. Science.

[28] Erb R.M., Libanori R., Rothfuchs N., Studart A.R. (2012). Composites Reinforced in Three Dimensions by Using Low Magnetic Fields. Science.

[29] Ding Q., Li X., Zhang D., Zhao G., Sun Z. (2019). Anisotropy of poly(lactic acid)/carbon fiber composites prepared by fused deposition modeling. J. Appl. Polym. Sci..

[30] Lu Y., Tong L. (2022). Concurrent optimization of topologies and fiber orientations for laminated composite structures. Compos. Struct..

[31] Reddy J.N. (2003). Mechanics of Laminated Composite Plates and Shells: Theory and Analysis.

[32] Talabi S.I., Chawla K., Rodriguez B., Barakat A., Meraki Y., Phadatare A., Brander M., Šeta B., Spangenberg J., Wu H.F. (2025). Fiber orientation and porosity in large-format extrusion process: The role of processing parameters. Compos. Part A Appl. Sci. Manuf..

[33] Lee Y., Lee S., Youn J., Chung K., Kang T. (2002). Characterization of fiber orientation in short fiber reinforced composites with an image processing technique. Mater. Res. Innov..

[34] Blanc R., Germain C., Costa J.P.D., Baylou P., Cataldi M. (2006). Fiber orientation measurements in composite materials. Compos. Part A Appl. Sci. Manuf..

[35] Jack D.A. (2006). Advanced Analysis of Short-Fiber Polymer Composite Material Behavior.

[36] Liu J., Wang Z., Zhao R., Wu T., Li Y., Li X., Yang T., Liang J. (2025). A composite material preparation method based on convergent die induced fiber orientation in DCCM process. Polym. Compos..

[37] Yeole P., Kim S., Hassen A.A., Kumar V., Kunc V., Vaidya U. (2021). Large-scale additive manufacturing tooling for extrusion-compression molds. Addit. Manuf. Lett..

[38] Bonati A., Pisano G., Royer Carfagni G. (2019). Redundancy and robustness of brittle laminated plates. Overlooked aspects in structural glass. Compos. Struct..

[39] Tian X., Liu T., Yang C., Wang Q., Li D. (2016). Interface and performance of 3D printed continuous carbon fiber reinforced PLA composites. Compos. Part A Appl. Sci. Manuf..

[40] Shirinbayan M., Nouira S., Imaddahen M.-A., Fitoussi J. (2024). Microstructure-sensitive investigation on the plastic deformation and damage initiation of fiber-reinforced polypropylene composite. Compos. Part B Eng..

[41] Hou Z., Tian X., Zheng Z., Zhang J., Zhe L., Li D., Malakhov A.V., Polilov A.N. (2020). A constitutive model for 3D printed continuous fiber reinforced composite structures with variable fiber content. Compos. Part B Eng..

[42] Shang J., Zhang W., Liu F., Wang S., Tian M., Ding X. (2023). Z-direction performance and failure behavior of 3D printed continuous fiber reinforced composites with sinusoidal structure. Compos. Sci. Technol..

[43] Shang J., Liu F., Zhang J., Chang B., Zhu C., Zhang W., Zhu Y., Ding X. (2025). Impact resistance of biomimetic gradient sinusoidal composites by 3D printing: Tunable structural stiffness and damage tolerance. Compos. Part B Eng..

[44] Liao G., Li Z., Cheng Y., Xu D., Zhu D., Jiang S., Guo J., Chen X., Xu G., Zhu Y. (2018). Properties of oriented carbon fiber/polyamide 12 composite parts fabricated by fused deposition modeling. Mater. Des..

[45] Caminero M.A., Chacón J.M., García-Moreno I., Rodríguez G.P. (2018). Impact damage resistance of 3D printed continuous fibre reinforced thermoplastic composites using fused deposition modelling. Compos. Part B Eng..

[46] Ming-Yuan H., Hutchinson J.W. (1989). Crack deflection at an interface between dissimilar elastic materials. Int. J. Solids Struct..

[47] Clementi G., Bonacci F., Caponi S., Cottone F., Gammaitoni L., Mattarelli M., Paccoia V.D., Perna G., Travasso F., Neri I. (2025). Energy loss mechanisms in foamed PLA cantilevers for low-vacuum applications. Smart Mater. Struct..

